# Prevalence and correlates of frailty in an older rural African population: findings from the HAALSI cohort study

**DOI:** 10.1186/s12877-017-0694-y

**Published:** 2017-12-28

**Authors:** Collin F. Payne, Alisha Wade, Chodziwadziwa W. Kabudula, Justine I. Davies, Angela Y. Chang, F. Xavier Gomez-Olive, Kathleen Kahn, Lisa F. Berkman, Stephen M. Tollman, Joshua A. Salomon, Miles D. Witham

**Affiliations:** 1 Center for Population and Development Studies, Harvard University, Cambridge, MA USA; 2 Medical Research Council/Wits University Rural Public Health and Health Transitions Research Unit, School of Public Health, Faculty of Health Sciences, University of the Witwatersrand, Johannesburg, South Africa; 3 School of Population Sciences and Health Services Research, Faculty of Life Sciences and Medicine, Centre for Global Health, King’s College London, London, UK; 4 Department of Global Health and Population, Harvard T.H. Chan School of Public Health, Boston, MA USA; 5 Epidemiology and Global Health Unit, Department of Public Health and Clinical Medicine, Umeå University, Umeå, Sweden; 6 Department of Global Health and Population, Harvard T.H. Chan School of Public Health, Boston, MA USA; 7 Ageing and Health, School of Medicine, University of Dundee, Scotland, UK; 8 INDEPTH Network, Accra, Ghana

**Keywords:** Fried frailty score, Mortality, Multimorbidity, Activities of daily living, Rural South Africa

## Abstract

**Background:**

Frailty is a key predictor of death and dependency, yet little is known about frailty in sub-Saharan Africa despite rapid population ageing. We describe the prevalence and correlates of phenotypic frailty using data from the Health and Aging in Africa: Longitudinal Studies of an INDEPTH Community cohort.

**Methods:**

We analysed data from rural South Africans aged 40 and over. We used low grip strength, slow gait speed, low body mass index, and combinations of self-reported exhaustion, decline in health, low physical activity and high self-reported sedentariness to derive nine variants of a phenotypic frailty score. Each frailty category was compared with self-reported health, subjective wellbeing, impairment in activities of daily living and the presence of multimorbidity. Cox regression analyses were used to compare subsequent all-cause mortality for non-frail (score 0), pre-frail (score 1–2) and frail participants (score 3+).

**Results:**

Five thousand fifty nine individuals (mean age 61.7 years, 2714 female) were included in the analyses. The nine frailty score variants yielded a range of frailty prevalences (5.4% to 13.2%). For all variants, rates were higher in women than in men, and rose steeply with age. Frailty was associated with worse subjective wellbeing, and worse self-reported health. Both prefrailty and frailty were associated with a higher risk of death during a mean 17 month follow up for all score variants (hazard ratios 1.29 to 2.41 for pre-frail vs non-frail; hazard ratios 2.65 to 8.91 for frail vs non-frail).

**Conclusions:**

Phenotypic frailty could be measured in this older South African population, and was associated with worse health, wellbeing and earlier death.

**Electronic supplementary material:**

The online version of this article (10.1186/s12877-017-0694-y) contains supplementary material, which is available to authorized users.

## Background

Frailty, conceptualised as the loss of ability to withstand a stressor [1], has emerged as a key construct in ageing and geriatric medicine over the last 15 years. In high-income countries, frailty has been shown to be an independent predictor of mortality, falls, length of hospital stay, functional decline, and need for social care [2–5]. Frailty also identifies the group of older people in higher-income countries most likely to benefit from Comprehensive Geriatric Assessment – a major healthcare intervention shown to improve outcomes in older people [6]. Frailty indices therefore have the potential to assist with identification of those at highest risk of these adverse outcomes, and hence target interventions to those most likely to benefit. Population-based studies on frailty can aid in identifying people at risk of frailty and the development of strategies for altering the trajectory towards frailty. As population ageing reaches across the world, preventive strategies to limit frailty and maximize healthy life expectancy will become increasingly important.

There are two main methods of identifying frailty – phenotypic scores and cumulative deficit scores. Phenotypic scores (e.g. the Fried frailty criteria [1]) focus on physical frailty and use a small number of measures – in Fried’s original paper, these were slow walk speed, low muscle strength, weight loss, self-reported exhaustion and self-reported low activity levels. In contrast, cumulative deficit models (e.g. Rockwood’s frailty index [7]) work by tallying the number of deficits across a wide range of organ systems – for instance diagnoses of disease, but also deficits on biochemical testing, cognitive test scores, and low measures of physiological function such as lung function or muscle strength. Such an approach is usually thought to require at least 30 variables for a reliable score [8].

Almost all work on frailty has taken place in older populations in high-income countries, characterised by well-funded, well-staffed, easily accessible healthcare services. Some work has been done in lower and middle-income countries, but this has mostly been confined to China, Mexico and Brazil [9]. The prevalence of frailty is very variable, both between countries, but also between populations within countries. A recent meta-analysis of Japanese studies showed a frailty rate of 7.4% in those aged 65 and over [10]. The prevalence in community-dwelling people in Europe and the USA was noted to be 10% in another recent analysis [11], but was higher in Latin America at nearly 20% [12]. A recent analysis using the frailty index approach suggested a lower prevalence of frailty amongst LMICs (Russia, China, South Africa, India, Ghana and Mexico) than in European countries [13]. This difference may be driven by methodological differences, but may also be due to survivor bias - in European countries, high quality health and social care systems may support those with frailty to live longer. A review of studies in LMICs, including Brazil, Mexico, China and Russia, suggested high rates of phenotypic frailty in community-dwelling older adults, from 17 to 44% depending on definition and population [14]. Given this disparity in prevalence rates between methods, settings and countries, data are required that are country-specific, using tools validated within the country or region of interest.

Populations in South Africa are now ageing at a rapid rate as a result of the scale-up of anti-HIV therapies [15]. There is consequently an urgent need to ensure that widely used frailty tools – which were developed elsewhere - are appropriate for use in a local population in order to inform the development of health and social care interventions and service delivery to maintain health and function in older age. Cumulative deficit frailty scores require collection of data on a large number of health domains – data that are not readily available in many healthcare systems in lower and middle income countries. In contrast, phenotypic frailty lends itself to measurement in both clinical and research practice even in resource-poor environments, as it does not require comprehensive sets of diagnoses or laboratory tests. In this paper, our objectives were to ascertain the prevalence of phenotypic frailty in a rural South African population of older people using a range of different ways of constructing a frailty phenotype score, to test whether phenotypic frailty was a distinct construct from disability and multimorbidity in this population, and to test whether frailty associated with earlier death and worse health and wellbeing in this population. We hypothesised that prevalence rates would differ from those seen in high income countries as a result of the above issues, that prevalence rates would vary depending on the ingredients of the frailty score, and that those individuals identified as being frail would have higher mortality rates, higher rates of impairment of Activities of Daily Living, and lower quality of life and general health than those who were non-frail. We also hypothesised that the group who were frail would be distinct from, but overlap with, those with disability and with multimorbidity.

## Methods

We used data from the Health and Aging in Africa: Longitudinal Studies of an INDEPTH community (HAALSI) survey of older people, conducted in the Agincourt subdistrict of rural northeast South Africa. As described previously [16], inclusion criteria for selection in the sampling frame were based on being permanently resident in the Agincourt HDSS area during the 12 months prior to the 2013 Agincourt Health and socio-Demographic Survey System (HDSS) census round [17], and aged 40 or over on July 1st 2014. Applying these criteria to the full 2013 Census data, produced a sampling frame of 8974 women and 3901 men aged 40 and older who met the residence criteria. Based on the assumption of an 80% response rate, we approached a total of 6281 people. Men were oversampled to ensure approximately even gender balance in the final consented sample. 5059 (85.9%) consented to participate in the HAALSI survey and provide the data for analysis in this paper. Participants unwilling or unable to consent to participate were excluded. Ethics committee approvals for HAALSI were obtained from the University of the Witwatersrand Human Research Ethics Committee (#M141159), the Harvard T.H. Chan School of Public Health Office of Human Research Administration (#13–1608), and the Mpumalanga Provincial Research and Ethics Committee.

All main survey data were collected in participants own place of residence. Data were collected by trained, local fieldworkers on laptops using Computer Assisted Personal Interviews (CAPI). Surveys were conducted in the local Shangaan language, with instruments translated from English and back-translated to ensure reliability. Measures of physical performance, blood tests and blood pressure were also conducted by the same fieldworkers, in the participant’s place of residence.

### Frailty score components

To measure walking speed, participants were asked to walk a 2.5 m course twice, with the time taken timed to the nearest 0.1 s. The course was marked out on flat, obstacle-free ground in or around the participant’s home. Participants were allowed to use any usual walking aids but no human assistance was permitted; participants started the course from a stationary standing position. The second walk was conducted on a course that was the reverse of the first course. Participants unable to conduct the walk test were assigned a walk speed of 0 m/s; those who declined to participate were omitted from analysis. The time for both walks was summed and a mean walk speed derived.

Grip strength was measured twice in both hands, using a Smedley digital dynamometer (12–0286). Testing was conducted with participants in a seated position, with the arm being tested held at 90 degrees of elbow flexion. For this analysis, the maximum reading achieved in either hand was used, as is the case in the majority of published cohorts [18]. Participants who were unable to perform grip strength testing because of pain or deformity in both hands, or other physical disability, were assigned a grip strength of 0 kg; no participant declined to participate in grip strength testing. Although no separate record was taken of those unable to complete grip testing due to cognitive impairment, most participants with missing grip strength data were able to complete other parts of the survey including cognitive testing, suggesting that cognition alone was unlikely to have prevented completion of grip strength testing.

Body mass index was derived from height measured to the nearest centimetre using an infra-red height sensor, and weight measured to the nearest 0.1 kg using the Genesis Growth Management Electronic Scale (Genesis; Johannesburg, South Africa). Physical activity was measured by self-report using the Global Physical Activity Questionnaire (GPAQ) [19]. Responses were used to calculate weekly activity in metabolic equivalents (METS). Sedentary time in hours per week was calculated from questionnaire responses; questions asked “how many hours did you spend sitting or reclining (excluding sleep) each weekday / weekend day”.

Self-reported exhaustion was measured using a positive response to a single question from the CES-D depression screening questionnaire: ‘much of the time you could not get going’ [20]. The original derivation of a frailty score by Fried et al. [1] used a positive response to either this question or a second question (‘everything you did was an effort’). Results from this second question could not be used due to ambiguities in the translation to the local Shangaan language. As an alternative to asking about exhaustion, we asked about change in overall health using the question ‘how has your overall health changed in the last 12 months’, with an answer of ‘worse’ or ‘much worse’ on a 5 point scale denoting a positive response. Whist this criterion differs from the original Fried criteria, the use of a question denoting decline over time is consistent with the vicious cycle of decline posited by Fried et al. as an underlying conceptual model for their frailty score.

Grip strength and walk speed were additionally adjusted for height as part of some frailty score variants. Regression analyses were run for males and females separately to calculate how grip and walk speed changed per cm increase in height. Both were normalised to the mean height (168 cm for men and 158 cm for women) of the HAALSI population; values of 0.29 kg per cm height (men) and 0.27 kg per cm height (women) were used to adjust grip strength; for walk speed, values of 0.0284 m/s (men) and 0.0392 m/s (women) were used.

### Calculation of frailty scores

We derived nine variants of a frailty score. Differences in available data between the original Fried derivation of frailty and the data available in HAALSI drove the choice of some components, but we also selected variants to explore the effect of height adjustment vs no height adjustment, and use of externally-derived thresholds vs use of lowest quintiles. Components of each score are depicted in the Additional file 1: Table S1. For each variant, we derived frailty classification in two ways: firstly, we included participants if at least three components were positive for frailty (frail) or three components were negative for frailty (non-frail). This allowed classification as non-frail or frail even if one or two components were missing in some cases. As this method did not allow classification as ‘pre-frail’ (1 or 2 components positive for frailty), we also derived classifications using only those participants with data on all components for a particular frailty score variant. We classified participants as non-frail (score 0); pre-frail (score 1 or 2) or frail (score 3 or more) for each frailty score as per Fried’s original derivation [1]. No weighting was applied to the components and no imputation was attempted for missing variables.

For some frailty score components, we selected external cutpoints based on previously published data. For low weight we used BMI < 18.5 kg/m^2^, a value widely used to define underweight [21]. For walk speed, we used <0.8 m/s, consistent with the value used to define sarcopenia in the European Sarcopenia Working group definition [22]. Male and female grip cutoffs are based on the 25th centile of grip found in healthy African populations aged 35–50 [23]. Additional file 1: Table S1 summarises the components of frailty scores that we considered, and the combinations of components that constituted the nine frailty score variants that we tested.

### Association with other constructs

To ascertain the utility of the scores in the local population, we tested the association between each frailty score and outcomes known to be associated with frailty, and also with variables measuring related but distinct constructs. Subjective wellbeing, self-reported health, impairment of basic activities of daily living (ADLs), and the presence of multimorbidity were assessed; methods for assessment are detailed in the Additional file 2. Date of death of individuals was ascertained during the 2016 HDSS census round (conducted between August and December 2016) by report from household members as previously described [17].

### Analyses

We analysed characteristics of the HAALSI population, prevalence of individual frailty components, and prevalence of frailty score categories for each frailty score variant. Overlaps between multimorbidity, disability (at least one impairment of basic ADLs) and frailty (score 3 or more) were depicted graphically. Wellbeing and general health were compared across frailty categories using ANOVA; the proportion of participants with at least 1 ADL impairment was compared across frailty categories using Pearson’s Chi-squared test.

Prediction of death by frailty category was performed using Cox regression, both unadjusted and adjusted for age and sex. Time to death from the date of first HAALSI interview was the dependent variable, and frailty category (non-frail, pre-frail, frail) for each frailty score was the predictor variable. Follow up was censored at the date which the individual was interviewed in the next HDSS census round, or the date of migration out of the Agincourt sub-district. A two-sided *p* value of <0.05 denoted significance for all analyses. Analyses were conducted using SPSS v24 (IBM, New York, USA).

## Results

A total of 5059 participants were recruited in HAALSI; baseline details for the HAALSI cohort are given in Table 1. Additional file 1: Table S2 shows the prevalence of each component in the HAALSI population, together with missing data rates. Missing data rates were low (<5%) for all components except BMI, number of sedentary hours per week, and height-adjusted grip and walk speed measures.

**Table 1:** Baseline details of the HAALSI cohort (*n* = 5059)

		Female	Male	All	P*
N (%)		2714 (53.6)	2345 (46.4)	5059	–
Mean age (years) (SD)		61.7 (13.3)	61.7 (12.8)	61.7 (13.1)	0.95
Age group (%)	40–49	500 (18)	418 (18)	918 (18)	<0.001
	50–59	785 (29)	625 (27)	1410 (28)	
	60–69	661 (24)	643 (27)	1304 (26)	
	70–79	432 (16)	446 (19)	878 (17)	
	80+	336 (12)	213 (9)	549 (11)	
Marital status	Never married	123 (5)	166 (7)	289 (6)	<0.001
	Divorced/separated	351 (13)	299 (13)	650 (13)	
	Widowed	1264 (47)	277 (12)	1541 (30)	
	Married/cohabiting	973 (36)	1601 (68)	2574 (51)	
HIV positive (%)		565 (21)	482 (23)	1047 (21)	0.82
Previous angina (%)		293 (11)	163 (7)	456 (9)	<0.001
Previous stroke (%)		63 (3)	86 (3)	149 (3)	0.31
Hypertension (%)		1806 (67)	1338 (57)	3145 (62)	<0.001
Diabetes mellitus (%)		324 (12)	235 (10)	559 (11)	0.03
Chronic bronchitis (%)		12 (0.4)	16 (0.7)	28 (0.6)	0.25
Anaemia (%)		1042 (38)	833 (36)	1875 (37)	0.15
Median 5 m walk speed (m/s) (IQR)		0.63 (0.50 to 0.83)	0.63 (0.50 to 0.83)	0.63 (0.50 to 0.83)	<0.001
Median maximum grip strength (Kg) (IQR)		22.5(17.7 to 27.3)	30.7 (23.2 to 37.2)	25.4 (19.3 to 32.3)	<0.001
Mean body mass index (Kg/m^2^) (SD)		29.3 (7.5)	24.9 (5.4)	27.3 (7.0)	<0.001
> = 1 basic ADL impairment (%)		257/2709 (9.5)	219/2332 (9.4)	476/5042 (9.4)	0.90

*ADL* Activity of daily living

*p for male vs female

**Fig. 1:**
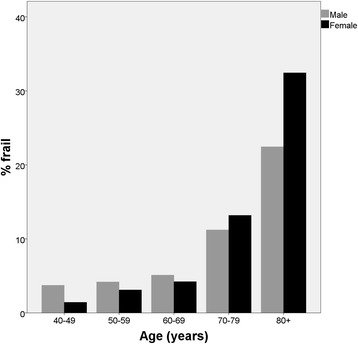
Differences in prevalence of frailty by sex and age category in HAALSI (frailty score variant 1)

Table 2 shows the prevalence of frailty and pre-frailty for each of the score variants. The prevalence of frailty was similar for most frailty score variants, but was markedly higher for those variants (6 and 7) using an unadjusted walk speed cutpoint of 0.8 m/s. Similarly, the prevalence of prefrailty was much higher, and the prevalence of non-frailty much lower, when using these two frailty score variants; the same finding was evident for the score variant (variant 9) using height-adjusted grip and walkspeed with externally derived cutpoints. Rates of non-calculable scores were unsurprisingly higher for all score variants when all five components were required to be present. Figure 1 shows that as expected, the prevalence of frailty rises with increasing age for both men and women, particularly over the age of 70 years.

**Table 2:** Prevalence of each category of frailty for all frailty score variants in HAALSI (*n* = 5059)

Frailty score variant	Allowing some missing data			Including only those with data on all 5 components			
	<3 (non-frail) (%)	3+ (frail) (%)	Not calculable (%)	0 (non-frail) (%)	1–2 (pre-frail) (%)	3+ (frail) (%)	Not calculable (%)
1	4477 (88.4)	372 (7.4)	210 (4.2)	2169 (42.9)	2117 (41.8)	228 (4.5)	545 (10.8)
2	4457 (88.1)	413 (8.2)	189 (3.7)	1949 (38.5)	2339 (46.1)	277 (5.5)	494 (9.8)
3	4486 (88.7)	337 (6.7)	236 (4.7)	1781 (35.2)	2308 (45.6)	238 (4.7)	732 (14.5)
4	4466 (88.3)	362 (7.2)	231 (4.6)	2097 (41.5)	2187 (43.2)	230 (4.5)	545 (10.8)
5	4561 (90.2)	288 (5.7)	210 (4.2)	2540 (50.2)	1822 (36.0)	153 (3.0)	544 (10.8)
6	4225 (83.5)	670 (13.2)	164 (3.2)	824 (16.3)	3304 (65.3)	487 (9.6)	444 (8.8)
7	4337 (85.7)	545 (10.8)	177 (3.5)	932 (18.4)	3312 (65.5)	372 (7.4)	443 (8.8)
8	4381 (86.6)	290 (5.7)	388 (7.7)	2018 (39.9)	2324 (45.9)	273 (5.4)	444 (8.8)
9	4322 (85.4)	337 (6.7)	400 (7.9)	888 (17.6)	3409 (67.4)	315 (6.2)	447 (8.8)

Table 3 shows the prevalence of disability (at least 1 ADL impairment), self-reported general health scores, and self-reported satisfaction scores across different levels of frailty for each score variant as a test of the construct validity of the frailty scores. For each score variant, participants in the frail category had worse outcomes in these domains than those in the non-frail category. A similar pattern was seen for scores calculated from all 5 components (Additional file 1: Table S3), where a clear gradient was evident from non-frail, to pre-frail and frail for each score variant. Individuals for whom a score could not be calculated had worse quality of life, worse general health and more ADL impairment than non-frail individuals. For score variants with more missing data, including scores calculated from all 5 components, ADL impairment rates were much higher in those with non-calculable scores than in those who were frail.

**Table 3:** Association between frailty score variants, wellbeing, self-reported health, and ADL impairment in HAALSI. Allowing some missing data

Frailty score variant		> = 1 ADL impairment (%)	Mean self-reported health score (SD)	Mean subjective wellbeing score (SD)
1	Non-frail	232/4466 (5.2)	2.22 (0.98)	6.87 (2.34)
	Frail	177/370* (47.8)	3.33* (1.14)	5.24* (2.49)
	Unable to calculate	67/206* (32.5)	2.81* (1.18)	5.93* (2.59)
2	Non-frail	241/4444 (5.4)	2.23 (0.98)	6.86 (2.35)
	Frail	180/410* (43.9)	3.25* (1.18)	5.49* (2.55)
	Unable to calculate	55/188* (29.3)	2.69* (1.14)	6.09* (2.66)
3	Non-frail	276/4473 (6.2)	2.24 (0.98)	6.85 (2.33)
	Frail	114/334* (34.1)	3.14* (1.23)	5.62* (2.77)
	Unable to calculate	86/235* (36.6)	2.87* (1.18)	5.90* (2.70)
4	Non-frail	228/4456 (5.1)	2.22 (0.98)	6.87 (2.34)
	Frail	170/359* (47.4)	3.27* (1.16)	5.25* (2.52)
	Unable to calculate	78/227* (34.4)	2.87* (1.19)	5.93* (2.62)
5	Non-frail	248/4549 (5.5)	2.23 (0.98)	6.85 (2.35)
	Frail	161/287* (56.1)	3.42* (1.14)	5.17* (2.56)
	Unable to calculate	67/206* (32.5)	2.87* (1.19)	5.91* (2.62)
6	Non-frail	207/4215 (4.9)	2.20 (0.97)	6.94 (2.32)
	Frail	223/665* (33.5)	3.04* (1.16)	5.36* (2.41)
	Unable to calculate	46/162* (28.4)	2.73* (1.13)	6.27* (2.62)
7	Non-frail	223/4328 (5.2)	2.22 (0.98)	6.90 (2.35)
	Frail	201/540* (37.2)	3.06* (1.17)	5.45* (2.40)
	Unable to calculate	52/174* (29.9)	2.82* (1.15)	6.15* (2.63)
8	Non-frail	239/4372 (5.5)	2.24 (0.99)	6.86 (2.35)
	Frail	70/286* (24.5)	2.83* (1.11)	5.78* (2.34)
	Unable to calculate	167/384* (43.5)	2.95* (1.27)	5.86* (2.72)
9	Non-frail	228/4313 (5.3)	2.23 (0.98)	6.88 (2.35)
	Frail	82/333* (24.6)	2.90* (1.08)	5.75* (2.37)
	Unable to calculate	166/396* (41.9)	2.92* (1.25)	5.86* (2.70)

*ADL* Activity of Daily Living

*p < 0.05 vs non-frail

There were 241 deaths during a mean follow up period of 17 months. Table 4 shows the results of Cox regression analyses comparing the hazard ratio for death during follow up for frailty strata in the different score variants; Additional file 1: Table S4 shows the same results using score variants calculated in those with all 5 components present. In unadjusted analyses, a clear gradient of risk of death was seen for all scores between non-frail, pre-frail and frail; the gradient was attenuated after adjustment for age, sex, comorbid disease, cognition, marital status and socioeconomic status, Whilst frailty category remained a strong predictor of death in each frailty score variant, some differences were evident between scores; score variants using a walk speed cutpoint of <0.8 m/s or those adjusting for height (variants 6,7,8 and 9) showed less difference in the risk of death between frail and non-frail individuals after adjustment for covariates. Score variants with more missing data tended to show lower hazard ratios for frailty, but higher hazard ratios for those for whom a frailty category could not be assigned.

**Table 4:** Hazard ratios for time to death for frailty categories in HAALSI. Allowing some missing data

Frailty score variant		Unadjusted hazard ratio (95% CI)	Adjusted hazard ratio (95% CI)
1	Non-frail	1 (−)	1 (−)
	Frail	7.66 (5.80 to 10.12)	4.12 (2.89 to 5.87)
	Unable to calculate	5.08 (3.38 to 7.63)	3.20 (1.89 to 5.40)
2	Non-frail	1 (−)	1 (−)
	Frail	7.20 (5.47 to 9.47)	3.85 (2.73 to 5.44)
	Unable to calculate	4.73 (3.06 to 7.30)	2.96 (1.73 to 5.07)
3	Non-frail	1 (−)	1 (−)
	Frail	6.21 (4.59 to 8.41)	3.48 (2.39 to 5.07)
	Unable to calculate	6.96 (4.96 to 9.77)	4.38 (2.89 to 6.65)
4	Non-frail	1 (−)	1 (−)
	Frail	8.28 (6.27 to 10.95)	4.15 (2.94 to 5.86)
	Unable to calculate	5.30 (3.59 to 7.84)	2.74 (1.64 to 4.57)
5	Non-frail	1 (−)	1 (−)
	Frail	8.91 (6.70 to 11.85)	4.63 (3.22 to 6.68)
	Unable to calculate	5.19 (3.48 to 7.73)	3.22 (1.91 to 5.44)
6	Non-frail	1 (−)	1 (−)
	Frail	5.62 (4.32 to 7.32)	3.15 (2.28 to 4.35)
	Unable to calculate	3.95 (2.38 to 6.55)	2.30 (1.20 to 4.44)
7	Non-frail	1 (−)	1 (−)
	Frail	6.13 (4.69 to 8.02)	2.68 (1.92 to 3.74)
	Unable to calculate	4.42 (2.79 to 7.00)	2.31 (1.26 to 4.23)
8	Non-frail	1 (−)	1 (−)
	Frail	2.78 (1.79 to 4.34)	1.78 (1.09 to 2.89)
	Unable to calculate	9.58 (7.31 to 12.55)	5.93 (4.36 to 8.26)
9	Non-frail	1 (−)	1 (−)
	Frail	2.65 (1.73 to 4.07)	1.45 (0.91 to 2.32)
	Unable to calculate	9.43 (7.19 to 12.36)	5.67 (4.06 to 7.91)

Adjusted for age, sex, hypertension, diabetes, stroke, chronic lung disease, anaemia, HIV, cognitive score, married/cohabiting vs single, and quintile of household wealth index

C-statistics using death during the 12 months following baseline assessment as the dependent variable were similar for each frailty score variant, with slightly higher c-statistics for score variants incorporating height adjustment. The combination of frailty score, demographic variables and comorbidity had high discriminatory value, with c-statistics between 0.80 and 0.85 for the different score variants as shown in Additional file 1: Table S5.

The Additional file 3: Figure S1 demonstrates the partial overlap between frailty, multimorbidity and disability for frailty score variant 1. Few participants were multimorbid, frail, and had ADL impairment, and only partial overlap was seen between frailty and ADL impairment despite relatively low numbers in both categories. Patterns of overlap were similar for all frailty score variants.

## Discussion

We have shown that variations of a phenotypic frailty score can be derived in this population of older rural South Africans, that these frailty score variants correlate with ADL impairment, poor self-reported general health and wellbeing, and that they independently predict all-cause mortality even after adjustment for comorbidity, cognition, demographic measures and socioeconomic measures. These results suggest that the frailty scores we derive measure a distinct construct to simply age or comorbid disease, and that they correlate with related constructs – a key component of validity. Although the precise measures in the scores that we derived differ slightly from the original criteria used by Fried et al., they were chosen to capture similar aspects to the underlying concept – a vicious cycle of decline – on which Fried and colleagues predicated their original frailty score. Each variant performed similarly; clear gradients of worsening self-reported health and subjective wellbeing with increasing frailty category were evident, rates of frailty were higher in women than in men at any given age as found previously [1], and rates of frailty rose steeply with age as expected. Furthermore, both frailty and pre-frailty predicted time to death over a mean 17 month follow-up, and ADL impairments were more common amongst the frail as compared to both pre-frail and non-frail. Importantly, although there was overlap between those with multimorbidity, frailty and disability, there was by no means complete congruence between these measurements. Current paradigms suggest that multimorbidity is a precursor to the development of frailty, and that frailty is a precursor to the development of disability [10]. Causal progression through these concepts is not inevitable however, with other factors (e.g. occult disease) contributing to frailty, and alternative pathways (e.g. a specific deficit such as a stroke) may lead to disability. Overlap between these concepts should therefore be incomplete, and our results support the fact that our frailty scores measure a concept distinct from both multimorbidity and disability. Our results suggest that the frailty construct that we are measuring behaves similarly to phenotypic frailty as measured in high income countries [1, 11], and that the ability of frailty to predict death in high-income countries also applies to older populations in this rural South African setting.

Our choice of frailty score components was necessitated in part by the measures included in the HAALSI survey, but also in part by logistical and cultural constraints. Quantifying weight loss for instance, was not possible as most older South Africans do not keep records of previous weight for comparison. Some questions for self-report (e.g. ‘everything is an effort’) did not translate easily to a similar concept in the population studied in this work and thus do not provide reliable data. Each population has characteristics of genetics, health behaviors, healthcare, comorbidity and ageing that are intimately linked to the particular countries and regions studied. It cannot therefore be assumed that existing frailty scores will automatically translate to low or middle income countries in other global regions, for instance sub-Saharan Africa. Furthermore, changes to the components included in phenotypic frailty scores can have significant impacts on the prevalence of frailty within a given population [11], thus any variation in how a frailty score is operationalised requires evaluation. Nevertheless, by choosing variables similar to those used by Fried et al., we aimed to preserve the concepts used by other phenotypic frailty scores even though the specific variables may differ slightly. The results from our analyses reinforce the need to derive appropriate thresholds for frailty score components in different populations; the very high prevalence of walk speed <0.8 m/s led to most of the study population being characterised as pre-frail; such an approach did not improve the strength of association between frailty scores and other outcomes. Similarly, a cutoff of <18.5 kg/m^2^ for BMI may not be appropriate for this population, and imposition of external cutpoints is a departure from the approach used by Fried et al. Use of a grip strength threshold derived from previous work in African populations was more successful, although such an approach was not superior to a quintile-based approach. Adjusting walk speed and grip for height weakened most associations studied; such an approach cannot therefore be recommended for this population as it adds to the complexity of measurement without adding to the usefulness of the frailty scores. As measured physical performance (grip strength, gait speed) in the HAALSI cohort was low by international comparison [12], the thresholds for deriving frailty components by quintiles in our study were very different from those used in high-income countries, reinforcing the need to use population-specific thresholds when operationalising frailty scores.

The similar performance seen for all the variants on a frailty score that we tested suggests that this is a robust approach to take, that the construct is able to tolerate multiple variations in its component parts, and that the construct measured by phenotypic frailty performs in South Africa in a similar way to in high-income countries (HICs). Our missing data rates for individual components of the frailty scores were low (0 to 7% of measures studied), and most people in the study population could be assigned a frailty category. The prevalence of frailty seen in this population was similar to that seen in some previous population-level studies; this is perhaps unsurprising given that for many of the components, the bottom 20% of the distribution was used to derive frailty, but similar prevalence figures were seen even when imposing externally derived cutpoints. Missing data may have led to an underestimate of the prevalence of frailty, as those with multiple missing datapoints are perhaps more likely to the frailest. This is borne out by the high hazard ratios for death, the high rates of ADL impairment and poor self-reported health in those for whom a frailty score could not be calculated. Inability to calculate a frailty score should therefore act as a marker for poor outcomes in the same way as frailty itself. Studies from other countries have shown a wide range of frailty prevalence, dependent on the age of those studied, as well as the group under study; those under medical care or in institutional care have much higher rates of frailty than those from general community-dwelling older populations. A recent meta-analysis of Japanese studies showed a frailty rate of 7.4% in those aged 65 and over [13]. Prevalence in community-dwelling people in Europe and the USA was noted to be 10% in another recent analysis [14], but was higher in Latin America at nearly 20% [24].

Comparing the prevalence of frailty between countries is thus fraught with difficulty. A recent analysis using the frailty index approach suggested a lower prevalence of frailty amongst LMICs (Russia, China, South Africa, India, Ghana and Mexico) than in European countries [25]. This difference may be driven by methodological differences, but may also (particularly for frailty index approaches) be due to less comprehensive diagnoses. If characterisation of frailty requires measurement and diagnosis across many body systems, failure to investigate and make diagnoses will tend to underestimate the prevalence of frailty. Another possible explanation is survivor bias - in European countries, high quality health and social care systems may support those with frailty to live longer. A review of studies in LMICs, including Brazil, Mexico, China and Russia, suggested high rates of phenotypic frailty in community-dwelling older adults, from 17 to 44% depending on definition and population [26]. It is important to note that the HAALSI population are younger (aged 40 and above) than those studied in the majority of HICs; the age-specific frailty prevalence within HAALSI is therefore higher than that seen in most previous studies in HICs, but is somewhat more similar to that seen in Latin America. This premature onset of frailty within LMIC populations is likely to pose important challenges for health and long-term care systems in LMICs.

Our study has a number of strengths. We used a large population-based sample; our results are therefore likely to be generalisable to a range of older South Africans living in rural environments. Our missing data rate was low, and the use of several score variants assists both with future efforts to implement such scores in practice, and with comparisons across populations. A number of weaknesses should also be highlighted. Our approach is slightly different from the original approach used by Fried et al. [1], which could have affected the prevalence of frailty that we found. However, the prevalence of frailty was similar across a range of scores utilising different components, including components used as an alternative to the exhaustion component of frailty, which we were unable to measure in the same way as Fried et al. in our cohort. Our data are derived from a research-based cohort study, not from clinical practice, and it remains to be seen how easy it is to implement these phenotypic scores in clinical practice, particularly in environments with constrained resources and few trained staff. Those in the youngest age group (40–49 year olds) are underrepresented in the HAALSI population in comparison to the whole HDSS population; a significant number of people in this age group are in work, often migrate for work, and hence fall outside the residency inclusion criteria that HAALSI employed in selecting the study population. The lack of long-term follow up data in the HAALSI project limits our ability to test how frailty scores predict onset of ADL limitation, though planned HAALSI follow-up surveys will allow for these analyses in future. Whilst the ability of frailty scores to predict hospitalisation and the need for institutional care is an important consideration in high-income countries, many older people in our study area do not have access to, or choose not to access, hospital services. Similarly, institutional care in our study area is extremely limited and this is thus a less relevant endpoint in this population.

How might phenotypic frailty scores be useful for future care and research? Firstly, the ability to measure frailty allows this to be used as a covariate in future analyses, particularly those examining the relationship between morbidity and outcomes. Secondly, the ability to identify frailty and pre-frailty will allow research on the determinants and consequences of frailty in this, and other sub-Saharan African populations. Ultimately identification of risk factors for frailty will permit the development of successful prevention strategies, and the ability to identify pre-frailty will potentially provide a window of opportunity for interventions to prevent progression to frailty. Within clinical practice, an easy to use frailty score will allow identification of those at risk of adverse health and functional outcomes, allowing planning of future health and social care needs at a population level.

## Conclusion

We have shown that phenotypic frailty scores can be derived in this older South African population, and that the components of such scores can be varied, albeit with some variation in measured prevalence of frailty. We have also demonstrated evidence of validity – frailty scores were associated with worse health, wellbeing and earlier death. Such scores will help to target interventions to prevent and treat frailty at an individual or community level – particularly comprehensive geriatric assessment, but also pleiotropic interventions (e.g. exercise and nutrition interventions) to delay the onset of adverse outcomes in older people. Whilst such interventions still require development and testing in sub-Saharan African populations, identification of those more likely to benefit is an important first step in addressing this issue, which will only increase in importance in the coming years.

## Additional files

Additional file 1: **Table S1.** List of components used to construct each frailty score variant tested. **Table S2.** Prevalence of, and correlations between, each frailty score component used from HAALSI. **Table S3.** Association between frailty score variants, wellbeing, self-reported health, and ADL impairment in HAALSI. **Table S4.** Hazard ratios for time to death for frailty categories in HAALSI. **Table S5.** Discrimination of different frailty score variants to predict death at one year. (DOCX 20 kb)

Additional file 2: Definitions of multimorbidity and other outcomes. Additional methods describing disease and multimorbidity definitions, activities of daily living and subjective wellbeing measures. (DOCX 16 kb)

Additional file 3: Figure S1. Overlap between prevalence of frailty, multimorbidity and impairment in Activities of Daily Living in HAALSI. (TIFF 121 kb)

## Abbreviations

ADL: Activities of Daily Living
CES-D: Centre for Epidemiological Studies – Depression scale
GPAQ: Global physical activity questionnaire
HAALSI: Health and Aging in Africa: Longitudinal Studies of an INDEPTH community
HDSS: Health and Demographic Surveillance Site
HIC: High income country
INDEPTH: International Network for the Demographic Evaluation of Populations and their Health
LMIC: Lower and middle income country
METS: Metabolic equivalents
