# Supplementary material for: Prevalence and correlates of frailty in an older rural African population: findings from the HAALSI cohort study
Source: BMC Geriatr. 2017 Dec 28;17:293. doi: 10.1186/s12877-017-0694-y (PMC5745732; doi:10.1186/s12877-017-0694-y)
Supplement: Supplementary file 1 — Table S1. List of components used to construct each frailty score variant tested. Table S2. Prevalence of, and correlations between, each frailty score component used from HAALSI. Table S3. Association between frailty score variants, wellbeing, self-reported health, and ADL impairment in HAALSI. Table S4. Hazard ratios for time to death for frailty categories in HAALSI. Table S5. Discrimination of different frailty score variants to predict death at one year. (DOCX 20 kb) [file 12877_2017_694_MOESM1_ESM.docx]

**Supplementary Table 1: List of components used to construct each frailty score variant tested**

|  | **Frailty sore variant** | | | | | | | | |
| --- | --- | --- | --- | --- | --- | --- | --- | --- | --- |
| **Component** | **1** | **2** | **3** | **4** | **5** | **6** | **7** | **8** | **9** |
| Lowest quintile of BMI  M<20.56kg/m^2^  F<23.34kg/m^2^ | **X** | **X** | **X** | **X** |  | **X** |  | **X** |  |
| Self-reported exhaustion (from a single CES-D question) | **X** |  | **X** | **X** | **X** | **X** | **X** | **X** | **X** |
| Lowest quintile of gait speed  M<0.46m/s  F<0.46m/s | **X** | **X** | **X** | **X** | **X** |  |  |  |  |
| Lowest quintile of grip strength  M<21.6Kg  F<16.4Kg | **X** | **X** | **X** |  | **X** | **X** |  |  |  |
| Worst quintile for weekly activity level by metabolic equivalents  M<30 MET mins/week  F<50 MET mins/week | **X** | **X** |  | **X** | **X** | **X** | **X** | **X** | **X** |
| Decline in general health over last 12 months |  | **X** |  |  |  |  |  |  |  |
| Worst quintile for  sedentary time  M>=42 hours/week  F>=39 hours/week |  |  | **X** |  |  |  |  |  |  |
| Grip <27kg (male)  Grip <13kg (female) |  |  |  | **X** |  |  | **X** |  |  |
| BMI<18.5kg/m^2^ |  |  |  |  | **X** |  | **X** |  | **X** |
| Walk speed <0.8 m/s |  |  |  |  |  | **X** | **X** |  |  |
| Lowest quintile of height adjusted walk speed  M<0.42m/s  F<0.38m/s |  |  |  |  |  |  |  | **X** |  |
| Lowest quintile of height adjusted grip  M<23.3kg  F<17.6kg |  |  |  |  |  |  |  | **X** |  |
| Height adjusted grip  <27kg (male)  <13kg (female) |  |  |  |  |  |  |  |  | **X** |
| Walk speed adjusted for height <0.8 m/s |  |  |  |  |  |  |  |  | **X** |

**Supplementary Table 2. Prevalence of, and correlations between, each frailty score component used from HAALSI**

| Component | Present(%) | Missing data / unable (%) | Overlap | | | | | | | | | | | | | |
| --- | --- | --- | --- | --- | --- | --- | --- | --- | --- | --- | --- | --- | --- | --- | --- | --- |
|  |  |  | Lowest quintile of BMI | Self-reported exhaustion | Lowest quintile of gait speed | Lowest quintile of grip strength | Worst quintile for weekly activity level by metabolic equivalents | Decline in general health over last 12 months | Worst quintile for  sedentary time | Grip <27kg (male)  Grip <13kg (female) | BMI<18.5kg/m2 | Walk speed <0.8 m/s | Lowest quintile of height adjusted walk speed | Lowest quintile of height adjusted grip | Height adjusted grip <27kg (male)  <13kg (female) | Walk speed adjusted for height <0.8 m/s |
| Lowest quintile of BMI  M<20.56kg/m^2^  F<23.34kg/m^2^ | 936 (18.5) | 371 (7.3) | - | 87 | 160 | 195 | 175 | 186 | 171 | 236 | 257 | 654 | 190 | 262 | 222 | 646 |
| Self-reported exhaustion (from a single CES-D question) | 433 (8.6) | 113 (2.2) | 87 | - | 140 | 120 | 122 | 133 | 104 | 131 | 37 | 357 | 100 | 106 | 92 | 281 |
| Lowest quintile of gait speed  M<0.46m/s  F<0.46m/s | 921 (18.2) | 233 (4.6) | 160 | 140 | - | 335 | 293 | 234 | 156 | 327 | 53 | 921 | 311 | 248 | 192 | 706 |
| Lowest quintile of grip strength  M<21.6Kg  F<16.4Kg | 1016 (20.1) | 0  (0) | 195 | 120 | 335 | - | 385 | 222 | 203 | 786 | 55 | 890 | 193 | 720 | 530 | 541 |
| Worst quintile for weekly activity level by metabolic equivalents  M<30 MET mins/week  F<50 MET mins/week | 1008 (19.9) | 12  (0.2) | 175 | 122 | 293 | 385 | - | 217 | 216 | 408 | 53 | 826 | 204 | 265 | 252 | 583 |
| Decline in general health over last 12 months | 928 (18.3) | 3  (0.1) | 186 | 133 | 234 | 222 | 217 | - | 201 | 263 | 60 | 697 | 176 | 213 | 187 | 600 |
| Worst quintile for  sedentary time  M>=42 hours/week  F>=39 hours/week | 893 (17.7) | 278 (5.5) | 171 | 104 | 156 | 203 | 216 | 201 | - | 239 | 41 | 648 | 158 | 183 | 171 | 556 |
| Grip <27kg (male)  Grip <13kg (female) | 1167  (23.1) | 0  (0) | 236 | 131 | 327 | 786 | 408 | 263 | 239 | - | 91 | 982 | 212 | 635 | 815 | 639 |
| BMI<18.5kg/m^2^ | 257  (5.1) | 371  (7.3) | 257 | 37 | 53 | 55 | 53 | 60 | 41 | 91 | - | 180 | 58 | 76 | 86 | 179 |
| Walk speed <0.8 m/s | 3677  (72.7) | 0  (0) | 654 | 357 | 921 | 890 | 826 | 697 | 648 | 982 | 180 | - | 908 | 771 | 705 | 2820 |
| Lowest quintile of height adjusted walk speed  M<0.42m/s  F<0.38m/s | 941  (18.6) | 365  (7.2) | 190 | 100 | 311 | 193 | 204 | 176 | 158 | 212 | 58 | 908 | - | 293 | 249 | 941 |
| Lowest quintile of height adjusted grip  M<23.3kg  F<17.6kg | 938  (18.5) | 365  (7.2) | 262 | 106 | 248 | 720 | 265 | 213 | 183 | 635 | 76 | 771 | 293 | - | 610 | 715 |
| Height adjusted grip <27kg (male)  <13kg (female) | 882  (17.4) | 365  (7.2) | 222 | 92 | 192 | 530 | 252 | 187 | 171 | 815 | 86 | 705 | 249 | 610 | - | 659 |
| Walk speed adjusted for height <0.8 m/s | 3298  (65.2) | 365  (7.2) | 646 | 281 | 706 | 541 | 583 | 600 | 556 | 639 | 179 | 2820 | 941 | 715 | 659 | - |

**Supplementary Table 3.** **Association between frailty score variants, wellbeing, self-reported health, and ADL impairment in HAALSI.**

Including only those with data on all 5 components

| **Frailty score variant** |  | **>= 1 ADL impairment (%)** | **Mean self-reported health score (SD)** | **Mean subjective wellbeing score (SD)** |
| --- | --- | --- | --- | --- |
| **1** | **Non-frail** | 58/2166 (2.7) | 2.04 (0.92) | 7.19 (2.25) |
|  | **Pre-frail** | 153/2113* (7.2) | 2.40* (0.99) | 6.57* (2.38) |
|  | **Frail** | 64/227** (28.2) | 3.07** (1.03) | 5.50** (2.38) |
|  | **Unable to calculate** | 201/536** (37.5) | 2.89** (1.24) | 5.90** (2.67) |
| **2** | **Non-frail** | 48/1946 (2.5) | 1.98 (0.87) | 7.20 (2.23) |
|  | **Pre-frail** | 170/2333* (7.3) | 2.43* (1.01) | 6.59* (2.39) |
|  | **Frail** | 72/277** (26.0) | 3.04** (1.11) | 5.84** (2.47) |
|  | **Unable to calculate** | 186/486** (38.3) | 2.84** (1.24) | 5.90** (2.67) |
| **3** | **Non-frail** | 54/1779 (3.0) | 1.99 (0.85) | 7.17 (2.23) |
|  | **Pre-frail** | 164/2300* (7.1) | 2.41* (1.04) | 6.70* (2.38) |
|  | **Frail** | 42/238** (17.6) | 2.89** (1.13) | 5.93** (2.73) |
|  | **Unable to calculate** | 216/725** (29.8) | 2.71** (1.21) | 5.96** (2.53) |
| **4** | **Non-frail** | 57/2094 (2.7) | 2.03 (0.92) | 7.18 (2.26) |
|  | **Pre-frail** | 151/2813* (5.4) | 2.40* (0.99) | 6.60* (2.36) |
|  | **Frail** | 67/229** (29.3) | 3.02** (1.05) | 5.48** (2.45) |
|  | **Unable to calculate** | 201/536** (37.5) | 2.89** (1.24) | 5.90** (2.67) |
| **5** | **Non-frail** | 62/2537 (2.4) | 2.04 (0.92) | 7.15 (2.28) |
|  | **Pre-frail** | 162/1817* (8.9) | 2.49* (1.01) | 6.45* (2.37) |
|  | **Frail** | 51/153** (33.3) | 3.13** (1.01) | 5.60 **(2.46) |
|  | **Unable to calculate** | 201/535** (37.6) | 2.89** (1.24) | 5.89 (**2.66) |
| **6** | **Non-frail** | 14/824 (1.7) | 1.91 (0.87) | 7.13 (2.24) |
|  | **Pre-frail** | 186/3297* (5.6) | 2.27* (0.98) | 6.90* (2.34) |
|  | **Frail** | 99/484** (20.5) | 2.83** (1.06) | 5.54** (2.35) |
|  | **Unable to calculate** | 177/437** (40.5) | 2.97** (1.26) | 5.85** (2.71) |
| **7** | **Non-frail** | 14/932 (1.5) | 1.93 (0.87) | 7.06 (2.29) |
|  | **Pre-frail** | 201/3305* (6.1) | 2.30* (0.99) | 6.85* (2.35) |
|  | **Frail** | 84/369** (22.8) | 2.83** (1.06) | 5.66** (2.32) |
|  | **Unable to calculate** | 177/436** (40.6) | 2.97** (1.26) | 5.84** (2.70) |
| **8** | **Non-frail** | 65/2015 (3.2) | 2.08 (0.95) | 7.13 (2.23) |
|  | **Pre-frail** | 169/2320* (7.3) | 2.37* (1.00) | 6.64* (2.43) |
|  | **Frail** | 65/270** (24.1) | 2.77** (1.08) | 5.78** (2.34) |
|  | **Unable to calculate** | 177/437** (40.5) | 2.97** (1.26) | 5.85** (2.71) |
| **9** | **Non-frail** | 19/888 (2.1) | 2.10 (0.93) | 6.82 (2.28) |
|  | **Pre-frail** | 207/3402* (6.1) | 2.26* (0.99) | 6.90 (2.36) |
|  | **Frail** | 73/312** (23.4) | 2.83** (1.05) | 5.74** (2.37) |
|  | **Unable to calculate** | 177/440** (40.3) | 2.96** (1.26) | 5.84** (2.69) |

ADL: Activities of Daily Living

***p<0.05 vs non-frail. **p<0.05 vs non-frail and vs pre-frail**

**Supplementary Table 4: Hazard ratios for time to death for frailty categories in HAALSI**

Including only those with data on all 5 components

| **Frailty score variant** |  | **Unadjusted**  **hazard ratio (95% CI)** | **Adjusted**  **hazard ratio (95% CI)** |
| --- | --- | --- | --- |
| **1** | **Non-frail** | 1 (-) | 1 (-) |
|  | **Pre-frail** | 1.53 (1.06 to 2.19) | 1.19 (0.82 to 1.74) |
|  | **Frail** | 5.17 (3.21 to 8.32) | 2.67 (1.57 to 4.54) |
|  | **Unable to calculate** | 8.97 (6.35 to 12.67) | 5.22 (3.45 to 7.88) |
| **2** | **Non-frail** | 1 (-) | 1 (-) |
|  | **Pre-frail** | 1.36 (0.94 to 1.96) | 1.03 (0.71 to 1.51) |
|  | **Frail** | 4.85 (3.07 to 7.64) | 2.19 (1.29 to 3.72) |
|  | **Unable to calculate** | 8.93 (6.26 to 12.75) | 4.72 (3.10 to 7.19) |
| **3** | **Non-frail** | 1 (-) | 1 (-) |
|  | **Pre-frail** | 1.58 (1.07 to 2.32) | 1.25 (0.84 to 1.87) |
|  | **Frail** | 4.47 (2.66 to 7.50) | 2.30 (1.28 to 4.13) |
|  | **Unable to calculate** | 7.09 (4.88 to 10.29) | 3.97 (2.60 to 6.05) |
| **4** | **Non-frail** | 1 (-) | 1 (-) |
|  | **Pre-frail** | 2.41 (1.62 to 3.59) | 1.78 (1.17 to 2.69) |
|  | **Frail** | 8.13 (4.96 to 13.35) | 3.92 (2.26 to 6.79) |
|  | **Unable to calculate** | 12.55 (8.48 to 18.58) | 7.16 (4.54 to 11.30) |
| **5** | **Non-frail** | 1 (-) | 1 (-) |
|  | **Pre-frail** | 1.80 (1.27 to 2.56) | 1.30 (0.90 to 1.88) |
|  | **Frail** | 6.03 (3.62 to 10.05) | 2.91 (1.65 to 5.12) |
|  | **Unable to calculate** | 9.24 (6.64 to 12.87) | 5.31 (3.57 to 7.91) |
| **6** | **Non-frail** | 1 (-) | 1 (-) |
|  | **Pre-frail** | 1.29 (0.79 to 2.11) | 1.12 (0.67 to 1.86) |
|  | **Frail** | 3.58 (2.06 to 6.24) | 2.18 (1.19 to 3.98) |
|  | **Unable to calculate** | 11.32 (6.90 to 18.57) | 6.72 (3.83 to 11.78) |
| **7** | **Non-frail** | 1 (-) | 1 (-) |
|  | **Pre-frail** | 1.94 (1.14 to 3.28) | 1.60 (0.92 to 2.77) |
|  | **Frail** | 4.92 (2.67 to 9.06) | 2.18 (1.12 to 4.23) |
|  | **Unable to calculate** | 15.30 (8.99 to 26.03) | 8.41 (4.62 to 15.29) |
| **8** | **Non-frail** | 1 (-) | 1 (-) |
|  | **Pre-frail** | 1.32 (0.93 to 1.87) | 1.13 (0.79 to 1.63) |
|  | **Frail** | 2.94 (1.76 to 4.93) | 1.91 (1.11 to 3.27) |
|  | **Unable to calculate** | 9.81 (6.98 to 13.79) | 6.06 (4.04 to 9.07) |
| **9** | **Non-frail** | 1 (-) | 1 (-) |
|  | **Pre-frail** | 1.94 (1.15 to 3.28) | 1.68 (0.99 to 2.85) |
|  | **Frail** | 4.46 (2.36 to 8.45) | 2.27 (1.17 to 4.40) |
|  | **Unable to calculate** | 14.70 (8.64 to 25.02) | 8.46 (4.77 to 15.00) |

Adjusted for age, sex, hypertension, diabetes, stroke, chronic lung disease, anaemia, HIV, cognitive score, married/cohabiting vs single, and quintile of household wealth index

**Supplementary Table 5. Discrimination of different frailty score variants to predict death at one year**

| **Frailty score variant** | **Harrell’s c statistic** | **95% CI** |
| --- | --- | --- |
| **1** | 0.82 | 0.78 to 0.86 |
| **2** | 0.83 | 0.79 to 0.86 |
| **3** | 0.83 | 0.79 to 0.87 |
| **4** | 0.83 | 0.79 to 0.87 |
| **5** | 0.82 | 0.78 to 0.86 |
| **6** | 0.82 | 0.78 to 0.86 |
| **7** | 0.82 | 0.78 to 0.85 |
| **8** | 0.85 | 0.81 to 0.88 |
| **9** | 0.84 | 0.81 to 0.88 |

All p<0.001

Score variants allowing some missing data used; all models included frailty score categorised as non-frail, frail or unable to calculate; age, sex, hypertension, diabetes, stroke, chronic lung disease, anaemia, HIV, cognitive score, married/cohabiting vs single, and quintile of household wealth index
