# Supplementary material for: Prevalence and correlates of frailty in an older rural African population: findings from the HAALSI cohort study
Source: BMC Geriatr. 2017 Dec 28;17:293. doi: 10.1186/s12877-017-0694-y (PMC5745732; doi:10.1186/s12877-017-0694-y)
Supplement: Supplementary file 2 — Definitions of multimorbidity and other outcomes. Additional methods describing disease and multimorbidity definitions, activities of daily living and subjective wellbeing measures. (DOCX 16 kb) [file 12877_2017_694_MOESM2_ESM.docx]

**S1. Definitions of multimorbidity and other outcomes**

*Determination of multimorbidity*

Multimorbidity was calculated based on the presence of nine chronic conditions, grouped into four categories of conditions: *cardiometabolic conditions* (hypertension, dyslipidaemia, diabetes, angina), *HIV*, *mental disorders* (depression, post-traumatic stress disorder, alcohol dependence), and *anaemia*. Individuals presenting with diseases in two or more of these categories of conditions were considered multimorbid; this approach avoids labelling individuals with a number of pathophysiologically related conditions (e.g. hypertension, angina, diabetes) as being multimorbid.

*Cardiometabolic disease conditions.* For both hypertension and dyslipidaemia, we applied the set of criteria listed in the South African and US guidelines [1-4]. Participants were considered hypertensive if systolic blood pressure was greater than or equal to 140 mmHg or diastolic blood pressure was 90 mmHg or higher, or if use of anti-hypertensive medication was reported at the time of interview. For dyslipidaemia, we classified those who meet at least one of the following criteria: total cholesterol >6.21 mmol/L, HDL-C <1.19 mmol/L, LDL-C >4.1 mmol/L, triglycerides >2.25 mmol/L, reported ever diagnosed with high cholesterol, or if use of medication is reported at the time of interview. Diabetes was diagnosed using the guideline published by the American Diabetes Association [5]: fasting glucose (defined as >8 hours) >7mmol/L (126 mg/dL) or non-fasting glucose level >11.0 mmol/L (200 mg/dL), reported ever being diagnosed with diabetes, or if use of medication is reported at the time of interview. Glucose was measured from a fingerprick blood sample using a CareSensN point of care glucose monitor (iSens Inc. Seoul, S Korea). Finally, angina was diagnosed by using the Rose Chest Pain Questionnaire, a widely used method in assessing angina [6].

*HIV****.*** For HIV, a positive diagnosis was recorded for individuals who tested positive for HIV via dried blood spot analysis using the Vironostika Uniform 11 assay (Biomerieux, France)

*Mental disorders.* Symptoms of depression were screened using the Center for Epidemiological Studies – Depression Scale (CES-D) 8-item questionnaire. We used a cutoff of three or more symptoms as a diagnosis of depression [7]. PTSD was diagnosed using a seven-symptom screening scale developed by Breslau et al [8], and individuals who score four or more on this scale were classified as having PTSD. Alcohol dependence was defined using the CAGE questionnaire, which includes four questions related to alcohol consumption [9].

*Others****.*** For anaemia, we measured haemoglobin using the point of care Hemocue system (Hemocue AB, Angelholm, Sweden). Anaemia was defined as a blood haemoglobin concentration of <13g/dL for men or <12g/dL for women, following the recommended cutoffs from the South African National Health and Nutrition Examination Survey [10].

*Other outcome measures*

Subjective wellbeing was assessed using the question ‘All things considered, how satisfied are you with your life as a whole these days?’. Respondents gave a reply from 0 to 10, where 0 was anchored as ‘dissatisfied’ and 10 was anchored as satisfied. Self-reported health was assessed using the question ‘In general, how would you rate your health today?’ Respondents answered on a five point scale, where 1 = very good, 3= moderate and 5= very bad. Disability was assessed by asking about the presence of impairment of basic activities of daily living (ADLs) (walking, transfers, toileting, bathing, eating and dressing) was assessed by self-report; for each activity, reporting difficulty in doing the activity, reporting inability to do the activity, or requiring help to do the activity constituted impairment of the ADL.

**References for Supplementary text**

1.Seedat YK, Rayner BL, Veriava Y. South African Hypertension Practice Guideline 2014 : Review Article. Cardiovasc J Africa. 2014;25:288–294.

2. National High Blood Pressure Education Program. 2004. “The Seventh Report of the Joint National Committee on Prevention, Detection, Evaluation, and Treatment of High Blood Pressure.”

3. Klug EQ, Raal FJ, Marais AD, et al. South African Dyslipidaemia Guideline Consensus Statement: A Joint Statement from the South African Heart Association (SA Heart) and the Lipid and Atherosclerosis Society of Southern Africa (LASSA). S African Fam Pract. 2013;55:9–18.

4. Expert Panel on Detection, Evaluation, and Treatment of High Blood Cholesterol in Adults. Executive Summary of the Third Report of the National Cholesterol Education Program (NCEP) Expert Panel on Detection, Evaluation, and Treatment of High Blood Cholesterol in Adults (Adult Treatment Panel III). JAMA. 2001;285:2486.

5. American Diabetes Association. Standards of Medical Care in diabetes—2015: Summary of Revisions. Diabetes Care 2015;38 (Suppl 1):S4

6. Rose, Geoffrey, P. McCartney, and D. D. Reid. 1977. “Self-Administration of a Questionnaire on Chest Pain and Intermittent Claudication.” British Journal of Preventive & Social Medicine 31 (1): 42–48.

7. Steffick DE. 2000. Documentation of Affective Functioning Measures in the Health and Retirement Study. Ann Arbor, MI: University of Michigan.

8. Breslau N, Peterson EL, Kessler RC, Schultz LR. Short Screening Scale for DSM-IV Posttraumatic Stress Disorder. Am J of Psychiatry. 1999;156:908–911.

9. Ewing JA. Detecting Alcoholism: The CAGE Questionnaire. JAMA 1984; 252: 1905–1907.

10. Shisana O, Labadarios D, Rehle T, Simbayi L, Zuma K. 2013. South African National Health and Nutrition Examination Survey (SANHANES-1). Cape Town HSRC Press.
