# Supplementary figures and images for: Prevalence and correlates of frailty in an older rural African population: findings from the HAALSI cohort study
Source: BMC Geriatr. 2017 Dec 28;17:293. doi: 10.1186/s12877-017-0694-y (PMC5745732; doi:10.1186/s12877-017-0694-y)

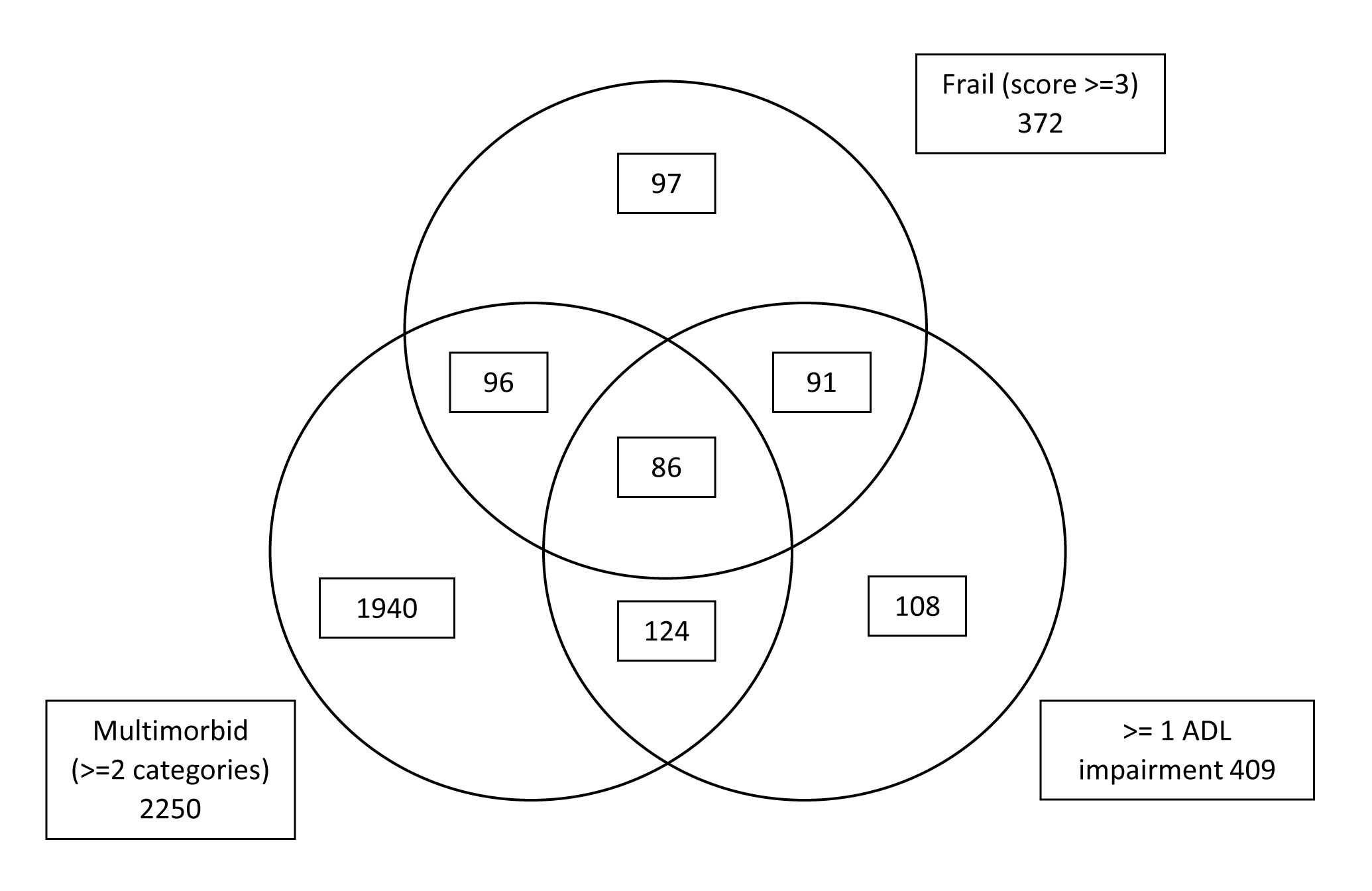

Supplement: Supplementary file 3 — Overlap between prevalence of frailty, multimorbidity and impairment in Activities of Daily Living in HAALSI. (TIFF 121 kb) [file 12877_2017_694_MOESM3_ESM.tif]
